# Effects of experimental sleep deprivation on aggressive, sexual and maternal behaviour in animals: a systematic review protocol

**DOI:** 10.1136/bmjos-2017-000041

**Published:** 2018-11-23

**Authors:** Gabriel Natan Pires, Andréia Gomes Bezerra, Rob B M de Vries, Cathalijn H C Leenaars, Merel Ritskes-Hoitinga, Sergio Tufik, Monica Levy Andersen

**Affiliations:** 1 Departamento de Psicobiologia, Universidade Federal de São Paulo, São Paulo, Brazil; 2 Department of Physiological Sciences, Santa Casa de São Paulo School of Medical Sciences, São Paulo, Brazil; 3 SYstematic Review Centre for Laboratory Animal Experimentation (SYRCLE) at Central Animal Facility, Radboud University Medical Center, Nijmegen, The Netherlands; 4 Institute for Laboratory Animal Science, Hannover Medical School, Hannover, Germany

**Keywords:** Bmjos, Meta-analysis As Topic, Animal Experimentation, Sleep Deprivation, Behavior

## Abstract

**Objective:**

Because of the relevance for the research on sleep deprivation and human behaviour, many preclinical studies have been conducted on aggressive, sexual and maternal behaviours in this field. Considering the available data and the complexity of the factors involved, the most appropriate way to summarise the effects of sleep deprivation on these behaviours is through systematic reviews and meta-analyses. This article describes the protocol for three independent systematic reviews and meta-analyses, evaluating the effects of sleep deprivation on aggressive, sexual and maternal behaviours in animals.

**Search strategy:**

A bibliographic search will be performed in four databases: Pubmed, Scopus, Web of Science and Psychinfo, searching for three domains: sleep deprivation (as the intervention), animals (as the population) and behaviour (as the outcome).

**Screening and annotation:**

Titles and abstracts will first be screened, followed by analysis of the full text and data extraction.

**Data management and reporting:**

SYstematic Review Centre for Laboratory Animal Experimentation ’s risk of bias tool will be used to evaluate risk of bias; visual analysis of funnel plots, Egger’s regression and trim-and-fill will be employed to evaluate publication bias. Effect sizes will be calculated from the articles by either direct or standardised mean difference, depending on the nature of the data. Overall estimates will then be calculated using a random effects model. Heterogeneity will be assessed using both I^2^ index and Cochran’s Q test. These meta-analyses should be useful to summarise the available data on the relationship between sleep deprivation and behaviour, providing a solid background for future behavioural sleep deprivation experiments, improving their validity.

## Introduction

Sleep deprivation is one of the most relevant and prevalent consequences of recent social changes and current lifestyle.^1–3^ Over the last six decades, total sleep time has reduced in about 2 hours per night.^4^ American samples show that 30% of the population sleep less than 6 hours per night and 40% reports less than 7 hours of sleep.^5 6^ The prevalence of individuals reporting less than 6 hours of sleep per night also seems to be increasing over the last three decades, in all age groups and both in men and women.^5^ Reports of insufficient sleep are consequently highly prevalent, ranging between 7% and 44% in different populations.^7–12^

Because of the high prevalence of sleep deprivation, multiple studies have been performed to evaluate its consequences. Among them, the effects on mental health are especially relevant.^13^ Sleep deprivation is related to neurobehavioural consequences and comorbidities, modifying and modulating behaviour in a broad manner.^14 15^ Some behavioural consequences of sleep loss, such as increased acute anxiety, lack of attention and aggressiveness, have already been reported since the classical article by William Dement.^16^ Since then, several studies have been conducted to evaluate how lack of sleep affects human behaviour.

Because of the relevance for human behaviour, many preclinical studies have been conducted to evaluate the effects of sleep deprivation, mainly in rodent models.^17^ Several animal models of sleep deprivation and behavioural assessment tools have been developed for use in animal experiments. These models and tools are employed to investigate the mechanisms underlying the relation between sleep deprivation and behaviour, the effect of drugs and other interventions and the adaptive behavioural mechanisms involved in this relationship.

The scientific output on sleep deprivation and behaviour in animals is prolific, with a strong emphasis on motivated social behaviours, such as aggressive, sexual and maternal behaviours. The association of sleep deprivation with these three behaviours has been the focus of research in human beings, providing reasonably consistent results. In short, lack of sleep has been associated with increased incidence of postpartum depression,^18–23^ increased aggressive behaviour and impulsivity,^24–30^ increased sexual arousal and decreased sexual motivation and satisfaction.^31–36^ In animals, sleep deprivation stimulates an externalising behavioural pattern. Individual studies show that sleep-deprived rodents are more aggressive,^24^ have more prominent sexual behaviour,^37^ display less depressive-like behaviour^38^ and present sustained levels of maternal behaviour.^39 40^ However, an overall and definitive behavioural phenotype of sleep-deprived rodents has not yet been described. A definitive behavioural phenotype is hard to achieve, given the variation in experimental design between animal behavioural studies, comprising, for example, species and strains, methods and duration of sleep deprivation and methods of behaviour assessment.

Considering the available amount of data and the complexity of the involved factors, the most appropriate way to summarise the behavioural effects of sleep deprivation is through systematic review and meta-analysis. This method allows us to comprehensively gather the studies published in this field, to reanalyze them altogether and to provide more information on the sleep–behaviour relationship. This approach has previously been used to analyse the effects of sleep deprivation on rodent anxiety-like behaviour.^41^ In summary, sleep deprivation leads to a condition of anxiolysis in rodents, opposite to the anxiogenesis observed in human beings. The anxiolytic effect is seriously affected by experimental factors such as the mode and duration of sleep deprivation and the method of behavioural evaluation.

Besides this analysis of anxiety-like behaviour, data on other sleep deprivation-related behaviours have never been comprehensively evaluated. We thus intend to use this approach to other behaviours in animals, first focusing on motivated social behaviours: aggressive, sexual and maternal behaviours. Our systematic reviews will determine the overall effect of sleep deprivation on these behaviours in animals, the impact of experimental variation on heterogeneity of the results and the animal-to-human translational potential of animal studies in this field. Furthermore, these systematic reviews will help to compose a behavioural profile of sleep-deprived animals, providing a solid background for further research within the wider field of sleep deprivation and behaviour. This specific article describes the protocol for our planned systematic reviews and meta-analyses.

## Methods

The sections below describe the protocol for three independent systematic literature reviews, addressing the effects of experimental sleep deprivation on aggressive, sexual and maternal behaviours. As these three systematic reviews are similar on their design, a common protocol is described below. Methods that apply specifically for one of the three reviews are clearly indicated, such as with the search strategy and inclusion and exclusion of studies. This protocol was prepared according to SYstematic Review Centre for Laboratory Animal Experimentation’s (SYRCLE) protocol for systematic reviews of animal studies.^42^

### Research question and basic definitions

These systematic reviews are intended to answer the following research question: What is the effect of experimental sleep deprivation on aggressive, sexual and maternal behaviours of healthy experimental animals, as compared with their controls. The reviews will focus on the following criteria:

*Disease/health problem of interest*: aggressive, sexual or maternal behaviours in animals.

*Intervention:* experimental sleep deprivation (encompassing total sleep deprivation, partial or stage-specific sleep deprivation, sleep restriction and sleep fragmentation).

*Control population:* non-sleep-deprived animals (encompassing separate animals in any type of control group (between-subject design) or the same animals at baseline prior to the intervention (within-subject design)).

*Outcome measures:* any behavioural variable of any test that evaluates aggressive, sexual or maternal behaviour in animals. A definitive list of behavioural tests will be available by the end of full-text screening.

### Bibliographic search

The search strategy was built according to Leenaars *et al* 43 for four different electronic databases: Pubmed, Scopus, Web of Science and Psychinfo. Despite not having a structured thesaurus-based searching system, we selected Scopus over EMBASE because of its wider coverage. While EMBASE comprises around 8500 journals, Scopus has over 33 000, including important sleep and neuroscience journals not found on EMBASE, such as *Nature and Science of Sleep* and *Sleep Science*. Web of Science includes regional databases such as SciELO, a Latin American scientific database, as well as other regional databases. As Latin America research plays an important role on sleep deprivation research, SciELO might bring additional studies that would otherwise not be retrieved. Psychinfo is the largest database for psychology. Many behavioural sleep deprivation studies are published in psychology journals, and some of them only indexed in Psychinfo.

The bibliographic search comprises three main domains: sleep deprivation (as the intervention), laboratory animals (as the population studied) and behaviour (as the condition of interest). It will first be created for Pubmed, and afterwards adapted to the syntax and search mechanisms of the other databases. For each one of the three planned systematic reviews, the search will be composed of a string for sleep deprivation, the SYRCLE animal filter^20^ and a string for the specific behavioural domain (aggressive, sexual or maternal).

Focusing on Pubmed, the string for sleep deprivation is composed of its Medical Subject Headings (MeSH) term ‘sleep deprivation’, as well as by other synonyms and related terms, not all included in the MesH. The string for behaviour encompasses the relevant MeSH terms, related terms, keywords, and methods used to measure the intended behaviour in animals. Items in the behavioural string may be truncated using the wildcard symbol ‘*’, to include all variations in both American and British spelling, as well as the plural and singular. In addition to the bibliographic search, reference lists of articles included will be analysed to retrieve additional papers. The Pubmed search strings are provided in box 1.

Box 1 **Pubmed search strings**

**Sleep deprivation:**

sleep deprivation[mesh] OR ((sleep[tiab] OR REM[tiab]) AND (depriv*[tiab] OR restrict*[tiab] OR fragment*[tiab] OR curtailment[tiab] OR loss[tiab] OR disrupt*[tiab] OR disturb*[tiab]))

**Animals: SYRCLE’s PubMed search filter^20^**

**Aggressive behaviour** 21–23 25:

aggression[mesh] OR violence[mesh] OR aggress*[tiab] OR agonistic behavio*[tiab] OR resident-intruder[tiab] OR tube test[tiab] OR territorial behavio*[tiab] OR dominance[tiab] OR social isolation[tiab] OR social provocation[tiab] OR violen*[tiab].

**Maternal behaviour** 21 26:

maternal behaviour[mesh] OR mother-child interaction[mesh] OR maternal behavio* OR maternal care[tiab] OR parental behavio*[tiab] OR parental care[tiab] OR mother-infant[tiab] OR maternal-infant[tiab] OR pup retriev*[tiab] OR nursing[tiab]

**Sexual behaviour** 21 27 28:

Sexual behaviour[mesh] OR animal sexual behaviour[mesh] OR sexual*[tiab] OR copulat*[tiab] OR mating*[tiab] OR courtship*[title] OR mount*[tiab] OR intromission*[tiab] OR ejaculat*[tiab] OR lordosis[tiab] OR genital*[tiab] OR erecti*[tiab]

### Study selection

Articles will be selected in two phases: first, title and abstract screening and second, full-text screening. The reference lists of included articles will be analysed and articles cited on these will also be included, if the inclusion criteria are met. Two independent reviewers will perform screening in both phases. Discrepancies in both phases will be resolved by discussion and consensus. If doubts persist, a third reviewer will be consulted.

### Inclusion and exclusion criteria

Articles will be evaluated according to the criteria described below.

#### Study design

Animal studies addressing sleep deprivation as intervention (independent variable) and aggressive, sexual or maternal behaviour as outcome (dependent variable) will be considered eligible. Sleep deprivation may be induced by experimental methods only, meaning that we will exclude studies of natural observation of lack of sleep, genetic models of insomnia and other non-experimental or non-induced sleep deprivation studies. Studies have to be controlled, either by having a separate control group (between-subject design) or by measuring the outcome at baseline before the sleep intervention (within-subject design). At least one sleep deprivation group or condition should be subjected only to sleep deprivation without concomitant intervention for inclusion. We will exclude articles lacking control groups or control conditions. Besides, we will exclude articles investigating the inverse relationship between our behaviours of interest and sleep (ie, addressing the effects of behavioural features on sleep), reviews, theoretical or non-original articles and articles for which full text is not available.

#### Animals

Only articles conduced with healthy experimental animals will be included. There are no restrictions to species, strain, sex or age. Articles conducted in unhealthy animals, and those in which a model of disease was induced, will be excluded.

#### Intervention

Articles using any type of sleep deprivation intervention (ie, any method, duration and frequency) will be included. Types of sleep deprivation comprise total sleep deprivation (complete absence of sleep), sleep restriction (reduction in total sleep time), partial sleep deprivation (deprivation of one sleep stage, such as rapid eye movement (REM) sleep and sleep fragmentation (intermittent awakenings through the sleep period). Articles featuring non-experimental sleep deprivation (natural observation of a lack of sleep, genetic models of insomnia, etc) will be excluded. Surgical or pharmacological methods of sleep deprivation will also be excluded, as they would lead to behavioural effects independent of sleep deprivation.

#### Outcome measures

Articles presenting any behavioural variable acquired through any test that directly or indirectly assess aggressive, sexual or maternal behaviour in animals will be considered eligible. Articles describing only other outcomes will be excluded.

#### Language and date restrictions

Articles published in any language will be considered eligible, provided that they are indexed in the aforementioned databases, that an abstract is provided in English and that full texts are available. Authors are fully able to evaluate and extract data from articles published in English, Portuguese, Spanish, French, Italian, Danish, German and Dutch. For articles in other languages, translators will be sought. There will be no restrictions to the date of publication.

#### Other criteria

Only articles published in peer-reviewed journals will be included; articles published in non-peer-reviewed sources will be excluded. If our searches retrieve duplicated or redundant data (ie, the same data were used on two or more articles), the duplicates will not be included. Unpublished data will not be considered.

In the title-abstract selection phase, exclusion criteria will be applied in the following order: (1) non-experimental article (reviews/theoretical articles); (2) not a study of healthy experimental animals (population); (3) no experimental sleep deprivation (intervention) and (4) no aggressive, sexual or maternal behaviours measured (outcome). In the full-text selection phase, exclusion criteria will be applied in the following order: (1) full text not available; (2) no control group or condition; (3) no experimental sleep deprivation and (4) no appropriate behavioural assessment.

### Data extraction

The included articles may well comprise several experiments. In preclinical sleep research, it is common to have two or more experimental groups compared with a single control group (eg, total sleep deprivation and sleep restriction). For our purposes, multiple experimental groups can be relevant, as sleep deprivation types are experimentally distinct and can lead to different results. Therefore, they cannot be grouped together and will be considered independently. The units of measurement for our analyses are the individual experiments, rather than the articles. In the meta-analyses, we will mathematically correct for the use of one control group in several experiments, as described in the Data synthesis and analyses section below. The variables to be extracted from each experiment are listed in table 1.

**Table 1:** Data extraction

Study design	Experimental groups and sample sizes
Animal model characteristics	Species, strain, sex and age. Data about oestrous phase will be extracted specifically for the review on sexual behaviour.
Intervention characteristics	Type of sleep deprivation, sleep deprivation method, time of sleep deprivation onset, duration of sleep deprivation protocol, recovery time between sleep deprivation and behavioural testing. Duration and onset of daily sleep opportunity time will be extracted in cases of studies employing sleep restriction protocols. Information about the timing of sleep deprivation (before pregnancy, during pregnancy or postpartum) will be extracted specifically for the review on maternal behaviour.
Outcome measures	Behaviour assessed, behaviour assessment method used, time and phase (light–dark cycle) of assessment and duration of behaviour analysis session. The exact postpartum day of behaviour analysis will be extracted specifically on the review about maternal behaviour.
Other data	Number of animals excluded (dropouts), reason for excluding animals and number of experiments per article.

Data extraction will be performed by a single reviewer and checked by a second one. Discrepancies will be resolved by consensus. For the behavioural outcomes, only numerical data will be extracted. Whenever an article provides only the SE of the mean, we will calculate the SD based on it and on the sample size. When needed, data will be estimated by graphs and figures using a digital ruler. There will be no restrictions concerning units of measurement. If an article does not fully describe the data or methods in detail, authors will be contacted and will be asked to provide their protocols, results or raw data. Specifically for cases in which the measure of variance used in error bars are not mentioned (SD vs SE of the mean), contacting authors will be the first approach, followed by assuming the bars as SD. After two unsuccessful contact attempts and if none of the options above is possible, the article can be excluded from specific analyses.

### Risk of bias and publication bias

Experimental bias affecting the included articles will be analysed using SYRCLE’s risk of bias tool,^44^ adapted for sleep deprivation studies as described before.^41^ In sleep deprivation studies, the experimental animals are usually submitted to sleep deprivation apparatuses or specific housing conditions, while control animals remain in their regular (or slightly modified) home cages. Consequently, group allocation is immediately recognised by housing and environmental characteristics and cannot be blinded. Besides, sleep-deprived animals are easily distinguished from control animals during observation and handling. Thus, the following items will be excluded from the risk of bias analysis: allocation concealment (selection bias) and blinding (performance bias).

Publication bias will be visualised with a funnel plot.^45^ The plot will depict the relation between the precision of each study (calculated as 1/√n) and its effects size.^46^ Additionally Egger’s regression and trim-and-fill analysis will also be performed to assess publication bias.

### Data synthesis and analyses

Independent analysis will be performed for each separate review focusing on the separate behaviours. A descriptive summary will be presented, starting with a flowchart of article selection and inclusion (as described by the Preferred Reporting Items for Systematic Reviews and Meta-Analyses statement^47 48^), followed by tables listing experiment characteristics. Meta-analyses will be performed if at least three comparable articles are retrieved.

Whenever all experiments in an analysis use the same behavioural test, the same unit of measurement and report the same animal model characteristics, intervention characteristics and outcome measures (as depicted in table 1), the effect size within our comparison will be a mean difference. Otherwise, the effect size within our comparison will be calculated using a standardised mean difference, based on Hedges’s G method. This latter method expresses the magnitude of the effect size in units of SD, thus allowing for comparisons of effects measured on different scales. Meta-analyses will use a random effects model (DerSimonian and Laird method). Heterogeneity will be assessed using both I^2^ index and Cochran’s Q test.

Depending on the nature of the data acquired, statistical corrections might be needed. First, whenever an article discloses a sample range instead of the exact number of animals per group, we will use the lower possible value in the provided range, avoiding any overestimation of effect. Sensitivity analyses will use the highest value from the sample ranges. Second, when two different experiments (or conditions) share a single control group (or baseline), the sample size of the control group will be adjusted by dividing it by the number of comparisons, avoiding overestimation of control group precision. Third, as multiple outcomes of a behaviour may be available per experiment, the best outcome will be chosen according to a list of the most frequently reported outcome measures.

To dissect possible sources of heterogeneity within the results, stratified subgroup analyses will be performed. Whenever there are more three or more comparable articles, these stratified analyses will be performed by species, strain, sex, age, type of sleep deprivation, length of sleep deprivation and method for behavioural assessment. Stratified and sensitivity analysis will also be performed according to research groups, in order to evaluate potential non-independence among results coming from a same groups of authors (‘research group bias’). We will perform stratified analysis specific to each research group whenever it has at least three articles included in our sample, as well as sensitivity analysis excluding a given research group from the pool whenever it contributed with at least 10% of the total sample. Research groups will be defined based on research group leaders. First, last and corresponding authors from the articles included in the analyses will be considered as potential research group leaders and will be ranked by number of entries. Each article will be accounted only once, being attributed to the research group of the highest-ranking of the author list. With equal ranks, the decision will prioritise the last author on a given article. A research group will only be defined if the potential research group leader has three or more entries in the ranking.

Data will be presented as effect size ±95% CIs. A p<0.05 will be considered significant on the primary meta-analysis. For stratified analysis, the p-value threshold for significance will be calculated with a Bonferroni correction, depending on the number of analyses to be performed. Results will be displayed in forest plots for primary analysis and as comparative bar charts for stratified analysis (as in Pires *et al* 41). Additional forest plots for the stratified analysis will be presented as supplementary material.

Finally, descriptive analyses will be performed to evaluate the temporal evolution and geographical distribution of articles included in our sample, separately for each of the three systematic reviews described. In each case, both general (encompassing all the articles included in each systematic review) and stratified analyses according to sleep deprivation methods, outcome assessment tool and species will be performed. These are descriptive analyses only; no statistical comparisons are planned. We will describe the temporal evolution of the number of articles on the topic of each review, as well as the countries that have contributed most.

## Discussion and expected results

An extensive amount of basic and preclinical sleep deprivation studies have been performed under a translational perspective, as a way to approach and dissect the effects of sleep loss on human health. Many of these animal studies evaluated the effects of sleep deprivation on behaviour, because neurobehavioural alterations and psychological burden are among the main deleterious effects of sleep loss in human beings. Data from 2010 estimate that more than 700 articles on the relation between sleep deprivation and behaviour in experimental animals are published each year.^49^

The amount of studies performed and published in this field could be manageable, as long as the studies have consistent and conclusive findings. However, this is lacking, due to high variability in both basic behavioural and clinical sleep deprivation research. Thus, a definitive behavioural profile of sleep-deprived animals is not yet available. Previous analyses of other behavioural outcomes, as well as the authors’ experience in the field, indicate that basic sleep deprivation studies have limited external validity. For example, a previous meta-analysis of the effects of sleep deprivation on anxiety-like behaviour has demonstrated that the effects observed in animals are the exact opposite of those observed in clinical settings, showing that lack of sleep leads to a decrease in anxiety-like behaviour in animals,^41 50^ while it leads to an increase in anxiety in humans.^51^ The same lack of replicability could occur for other behaviours, such as those described in the current protocol.

Another concern with the use of animal models for sleep deprivation experiments is animal welfare.^52^ Both spontaneous and experimental sleep deprivation always coincide with a stress response. This welfare concern would be acceptable, as long as the sleep deprivation studies provide reliable results.^52^ If our meta-analyses show that the animal studies have limited external validity, new experiments in this field should be discouraged (as for anxiety-like behaviours).

The scientific output on the relation between sleep deprivation and aggressive, sexual and maternal behaviours have been piling up for years, without a comprehensive review. A broad reanalysis as described in this protocol can provide new guidance to the field, allowing future research on sleep and behaviour to develop on a firm and less biased basis. Our reviews will have the following practical benefits.

*Overall estimate of effect of sleep deprivation on behaviour:* the generic aim of the analyses here proposed is to determine the effect of different types and methods of sleep deprivation on aggressive, sexual and maternal behaviour.

*Profile of scientific output in the field:* these reviews will show the profile of the scientific publications in the field, including the temporal evolution. We can then analyse which countries and research groups account for the majority of the output. Also, we can evaluate which models of sleep deprivation and which animal species have been used and how this evolved. For example, the use of *Drosophila* and zebrafish for sleep deprivation studies became increasingly common over the last decade, but so far, there is no evidence that it has led to a decreased use of rodents.

*Translational reliability of sleep deprivation:* once the meta-analyses are performed, it will be possible to evaluate how reliable animal studies are, compared with data raised in clinical settings. One of the most important principles of translational biomedical research is to provide knowledge that can be applied in clinical science. If the behavioural effects of sleep deprivation are opposite or somewhat different to those observed in humans (as for anxiety), research in this field should be interpreted cautiously, as the translational value might be limited. On the other hand, if behavioural effects of sleep deprivation are comparable between animals and humans, we may carefully extrapolate mechanistic knowledge gained from animal studies to clinical sciences.

*Optimisation of experimental design:* it is quite likely that studies using certain species, sleep deprivation techniques or behavioural measurement tools provide better and more consistent results than others, and even that they are more reliable from a translational perspective. Certain methods can be more sensitive to detect translational outcomes under sleep-deprived conditions and should thus be first choice for future experiments. Methods that provide inconsistent, unreliable or opposite results should be avoided.

*Behavioural profile of sleep-deprived animals:* each meta-analysis will evaluate the effect of sleep deprivation on a single behaviour. However, sleep deprivation is a complex intervention, resulting in a specific and multifaceted behavioural phenotype. Taken altogether, these three meta-analyses will compose a behavioural profile of sleep-deprived animals, specifically for motivated social behaviours.

*Reduce redundant, duplicated or unnecessary studies* it is natural and useful in biomedical sciences to perform studies replicating previous results, in order to confirm data. However, unnecessary superfluous replication is common to animal experiments. Experiments conducted after a finding has proven reliable and consistent are redundant and do not contribute to the whole body of knowledge, being a waste of time, efforts and animal resources. By means of meta-analyses, we can identify if the research on sleep and aggressive, sexual and maternal behaviours has already reached reliability and consistency, in which case further experiments will no longer be necessary.

*Background for future sleep-deprivation studies*: complementary to the previous item, systematic reviews and meta-analyses are useful to identify knowledge gaps and indicate which experiments are still needed, because of inconclusiveness, inconsistency of the results or lack of data. For example, the aforementioned meta-analysis of sleep and anxiety-like behaviour identified that the amount of data for total sleep deprivation and sleep restriction is sufficient to provide definitive conclusions, but that overall data are still inconclusive for sleep fragmentation, for which more experiments are actually still needed. Thus, by identifying for which protocols data are conclusive and for which they are not yet, we can provide a thorough justification for new experiments.

The benefits listed above indicate that systematic reviews of animal data can be linked to the ethics of animal experiments. Systematic reviews emerged as a way to summarise animal data in a rigorous way, which allows for evaluation of the translatability of animal data into clinical settings.^53–55^ As they can also prevent the unnecessary use of animals, systematic reviews became an important tool in the field of animal welfare and ethics in animal experimentation. Systematic reviews can inform on all the three R principles (replacement, reduction and refinement) proposed by Russel and Burch.^56^ First, by avoiding redundant replication of experiments, they lead to the reduction of the total number of animals used. Second, by analysing which species, protocols and measurement tools are most sensitive to detect the outcomes of interest, they promote the refinement of the animal experiments. Third, as meta-analyses can be used to address new research questions and to increase the power of previously performed separate studies they can be considered an alternative method themselves.

In parallel with the strengths on the planned systematic reviews and meta-analysis, a few limitations should be acknowledged beforehand. Despite the number of articles on the relationship between sleep deprivation and animal behaviour in general being high, we are aware that this might not be reflected for each specific behaviour. Thus, we expect a variation on the number of included records between the three planned meta-analyses, which will influence the conclusive potential in each case. Additionally, behavioural animal studies are known for their diversity in experimental design and for their low replicability. Consequently, data heterogeneity and inconsistencies on the results might be an important drawback for the analyses. Of note, preclinical meta-analyses do not intend to generate firm conclusions based on effect estimates. Rather, they aim to explore data within a field of research, understanding the impact of different confounders, methods and experimental protocols, analysing which provide more consistent results and which do not seem to provide reliable data. Knowing how many studies have been done on the relationship between sleep deprivation and aggressive, sexual and maternal behaviours is a result in itself. Likewise, evaluating the consistency on the results and exploring which methods have been successfully employed is an important way to avoid duplicated or redundant experiments in the future.

Based on previous experiments, our hypothesis is that sleep deprivation leads to increased aggressive behaviour, decreased sexual and sustained maternal behaviour. We can only evaluate if this hypothesis is true by means of the proposed meta-analyses. Moreover, only by these analyses we will be able to see if results are consistent between different species, types of sleep deprivation and behavioural assessment tools. The planned meta-analyses should be useful to summarise the available data on the relationship between sleep deprivation and motivated social behaviour. The summaries will benefit future research in the field and improve the use of animal resources in behavioural sleep deprivation experiments.
